# Effect of cutting management on the forage production and quality of tepary bean (*Phaseolus acutifolius* A. Gray)

**DOI:** 10.1038/s41598-023-39550-3

**Published:** 2023-08-08

**Authors:** Travis W. Witt, Brian K. Northup, Timothy G. Porch, Santos Barrera, Carlos A. Urrea

**Affiliations:** 1 USDA-ARS, PA, Oklahoma and Central Plains Agricultural Research Center, 7207 West Cheyenne Street, El Reno, OK 73036 USA; 2 USDA-ARS, Tropical Agriculture Research Station, 2200 P.A. Campos Ave., Suite 201, Mayagüez, 00680 Puerto Rico; 3 Department of Agronomy and Horticulture, University of Nebraska, Lincoln, NE 68583 USA; 4 University of Nebraska-Lincoln, Panhandle Research and Extension Center, 4502 Avenue I, Scottsbluff, NE 69361 USA

**Keywords:** Plant sciences, Natural variation in plants, Plant domestication

## Abstract

Tepary bean (*Phaseolus acutifolius* A. Gray) is an underutilized drought tolerant annual legume, originating from the Sonoran Desert, that may be a beneficial forage/hay for beef cattle in the Southern Great Plains of the US (SGP). The SGP has erratic rainfall and periods of intermittent drought exacerbated by high summer temperatures. In 2020 and 2021, a split-plot design was used to evaluate 13 genotypes of tepary bean and a forage soybean (control) at El Reno, OK, USA to compare production of plant biomass and forage nutritive value parameters under seven harvest regimes. Genotypes were used as the main plot and cutting management as the sub-plot. Biomass production of all tepary bean genotypes equaled that of soybean (*p* > 0.05), while several genotypes had superior forage nutritive value traits (*p* ≤ 0.05). Overall, a 15-cm cutting height and 30-day harvest interval produced the best overall product (average dry biomass of 5.8 Mg ha^−1^ with average relative feed values (RFV) of 165). Although all harvest regimes reduced total seasonal biomass, forage nutritive value increased. However, the tradeoff between forage production and nutritive value may be unacceptable to most producers. Further agronomic and breeding research is needed to encourage producers to grow tepary bean as a forage/hay in the SGP.

## Introduction

The states of Kansas, Oklahoma, and Texas in the US Southern Great Plains (SGP) had over 24.4 million head of cattle as of January 2022^1^. Traditionally warm-season perennial grasses are used to support cattle in the SGP; however, all nutritive value characteristics of these forages decrease with maturation in July through September^2^. The decrease in forage nutritive value is due to many plant and environmental factors, such as solar radiation, photosynthesis, growth patterns, and temperatures^3^. Additionally, the nutritive value of perennial legumes such as alfalfa also decline during the summer^4^, and alfalfa can be difficult to establish and maintain in dryland environments. Currently, many producers use annual legumes to provide forages with high nutritive value as alternatives to perennial grasses.

The taproot architecture of their roots allows legumes to access subsoil nutrients and moisture that is often unavailable to grasses^5^. Tepary bean (*Phaseolus acutifolius A. Gray*) is an underutilized drought tolerant legume that has been grown for thousands of years within its natural range, which encompasses an area from Arizona, USA to Guatemala^6,7^. However, tepary bean was not improved using modern breeding techniques until 2013 and then only for grain production^8^. Ongoing research suggests that tepary bean is well suited to growing conditions in the SGP and can produce similar forage biomass production and nutritive value as soybean, but with less water^9–11^. Additionally, tepary bean performed as well or better than cowpea (*Vigna unguiculata*), lablab (*Lablab purpureus* L.), and soybean (*Glycine max*) in legume/forage sorghum mixtures for the traits of acid detergent fiber and crude protein during the early growing season^12^.

Given the historical use of tepary bean as a grain legume, the impact of harvest management (height and frequency) on biomass production and forage nutritive values is unknown. This information is important for legumes because defoliation causes a cessation of nitrogen fixation^13^, and harvesting close to the ground can remove the meristematic tissues on stems that are required for regrowth. The objective of this study was to compare and evaluate 13 tepary bean genotypes and one soybean control for forage biomass production and nutritive value under rainfed conditions in the SGP. Our hypothesis was that cutting management would reduce the biomass production and nutritive value of tepary bean.

## Materials and methods

### Site information

On June 10, 2020 and 2021, ten genotypes of tepary bean and one genotype of forage soybean (‘Laredo’) were planted in a split-plot experimental design as the main plot, arranged in a randomized complete block design with three replications at the Oklahoma and Central Plains Agricultural Research Center, El Reno, OK (35° 34′ N; 98° 2′ W, 414 m a.s.l.). Blocks each year helped minimize variations in availability of soil nutrients for the two soil types. Laredo soybean was chosen as the control for its’ availability to producers in the SGP. Due to a lack of seed, three genotypes of tepary bean were replaced in 2021 (*n* = 11 each year; 10 tepary beans and 1 soybean). Table 1 describes the genotypes used in the study.

**Table 1:** Tepary genotypes and forage soybean control grown at El Reno, OK 2020 and 2021.

Genotype	Species	Type	Source	Year tested
PI 310800	*P. acutifolius*	Landrace	Chinandega, Nicaragua	2020
PI 440802	*P. acutifolius*	Landrace	Arizona, US	2020
Black	*P. acutifolius*	Variety	Native Seeds Search, Arizona, US	2020 & 2021
G40068	*P. acutifolius*	Landrace	Arizona, US	2020 & 2021
G40119	*P. acutifolius*	Landrace	Oaxaca, Mexico	2020 & 2021
G40173A	*P. acutifolius*	Landrace	Sonora, Mexico	2020 & 2021
G40200	*P. acutifolius*	Landrace	Guanacaste, Costa Rica	2020 & 2021
G40284	*P. acutifolius*	Landrace	Arizona, US	2020 & 2021
‘Laredo’ Soybean	*Glycine max*	Cultivar	Ross Seed Company, Oklahoma, US	2020 & 2021
TARS-Tep 4	*P. acutifolius*	Breeding line	USDA-ARS, Puerto Rico	2021
TARS-Tep 6	*P. acutifolius*	Breeding line	USDA-ARS, Puerto Rico	2021
TARS-Tep 10	*P. acutifolius*	Breeding line	USDA-ARS, Puerto Rico	2021
TARS-Tep 22	*P. acutifolius*	Germplasm	USDA-ARS, Puerto Rico	2020 & 2021
TARS-Tep 23	*P. acutifolius*	Germplasm	USDA-ARS, Puerto Rico	2020

The plots were 1.4 m long and 1.0 m wide with 0.25 m between rows. Each plot was sub-divided into 0.5 m × 0.25 m areas for data collection with cutting height and interval (cutting management) applied at fixed locations within each plot. Tepary beans were planted in four-row plots at a rate of 57 seeds per meter. Soils at the site were members of the Brewer silty clay loam loam (fine, mixed, superactive, thermic Pachic Udertic Argiustolls) series in 2020 and the Dale silt loam (fine-silty, mixed, thermic Pachic Haplustolls) series in 2021. Within the top 15 cm, the Brewer silty clay loam has a pH of 6.5 and the Dale silt loam has a pH of 6.1^14^. The Brewer silty clay loam has 30% clay, 18% sand, and 52% silt and carbon and nitrogen values of 1.3 ± 0.3% and 0.1 ± 0.03% in the upper 30 cm. The Dale silt loam has 20% clay, 11% sand, and 69% silt and carbon and nitrogen values of 1.1 ± 0.2% and 0.1 ± 0.02%. Rainfall during the growing season was 340 mm in 2020 and 271 mm in 2021. Weedy grasses were controlled with Clethodim 2EC throughout the growing season at a rate of 231.5 g a.i. ha^−1^).

### Data collection

Biomass was harvested from 0.5 m row lengths at 30-, 45-, or 90-day (end of season) intervals and at heights of 5, 10, or 15 cm above ground level to determine biomass production and regrowth ability. The 90-day interval was cut at 5 cm only and served as a control. Fresh weight of biomass was determined for clipped samples which were then dried at 65 °C for 72 h, re-weighed to define dry matter, and ground to a 2.0 mm particle size for laboratory analysis (Thomas Scientific Wiley Mill, Swedesboro, NJ, USA). The ground particles were thoroughly mixed and ~ 50 g were scanned with a benchtop NIR (Unity Scientific Spectra Star XT with UCal calibration software, Westborough, MA, USA). Measures of forage nutritive value [acid detergent fiber (ADF), in vitro True Digestibility (IVTD), neutral detergent fiber (NDF), total nitrogen content (N), total digestible nutrients (TDN), and a TDN:CP ratio] were defined from scans. The benchtop NIR was validated with wet chemistry each year using approximately 10% of the samples. Occasionally, due to reduced plant growth, replications were combined to determine the forage nutritive value of a genotype.

After determining percent N in samples, crude protein was calculated using the formula: CP = N% × 6.25^15^. Relative feed values (RFV) were calculated from ADF and NDF measurements with the formula: RFV = DDM x DMI ÷ 1.29, where DDM (digestible dry matter) = 88.9 − (0.779 × %ADF) and DMI (dry matter intake) = 120 ÷ %NDF^16^. Total digestible nutrients (TDN) were determined from ADF values using the formula: TDN_legume_ = 88.875 − (0.812 × %ADF). The TDN:CP ratio was calculated using the formula TDN:CP = TDN ÷ CP.

### Data analyses

The biomass harvested at each date within a plot were added together to represent the total annual biomass (i.e., the three 30-day and two 45-day cuttings were summed within management (interval) and compared to the 90-day cutting). Measures of forage nutritive values from each harvest date within a plot were multiplied by the respective biomass, added together, and divided by total biomass to derive annual measures of nutritive values based on weighted averages. Data were analyzed with the Proc GLIMMIX procedure in SAS Studio 3.8^17^. The following model was used: Y_ijkl_ = μ + management_i_ + genotype_k(i)_ + year_j_ + managementxyear_ij_ + Error_k(ij)_. Genotype, cutting management (cutting height and cutting interval), and their interactions were considered fixed effects while the intercept of the linear predictor was considered a random effect with year as the subject (level). Mean separation and determination of least significant differences were evaluated using the Tukey adjustment or Tukey–Kramer adjustment for unbalanced designs. When data did not follow a normal distribution (i.e., percentages), the link/ilink functions (link the data scale to the model scale) were used^17,18^. Additional information on the analyses of variance (ANOVA) can be found in Table 2.

**Table 2:** P-values of analysis of variance (ANOVA) of fixed main effects and interactions.

	Genotype	Management	Genotype × Management
	p-value		
ADF	0.26	< 0.01	0.80
Biomass (dry)	0.01	< 0.01	0.62
CP	< 0.01	< 0.01	0.78
IVTD	0.32	0.08	0.12
NDF	< 0.01	< 0.01	0.33
RFV	< 0.01	< 0.01	0.25
TDN	0.46	0.06	0.18
TDN:CP	< 0.01	< 0.01	0.77

ADF is acid detergent; CP is crude protein; IVTD is InVitro true digestibility; NDF is neutral detergent fiber; RFV is relative feed value; TDN is total digestible nutrients; TDN:CP is the ratio of total digestible nutrients to crude protein.

### Research involving plants

All plant collections were done in accordance with international law. Seed is available to reproduce the experiment from USDA/ARS/GRIN (https://www.ars-grin.gov/).

## Results

### Genotypic by management effect

Main effects of genotype and cutting management affected overall production of biomass and forage nutritive value. However, no traits had a significant genotype by management interaction (p > 0.05). This indicates that all the genotypes responded similarly to the different forms of applied management. The lack of diversity in response of tepary bean to cutting management will make breeding efforts to improve tolerance to repeated cutting difficult.

### Genotypic effect

**Figure 1:**
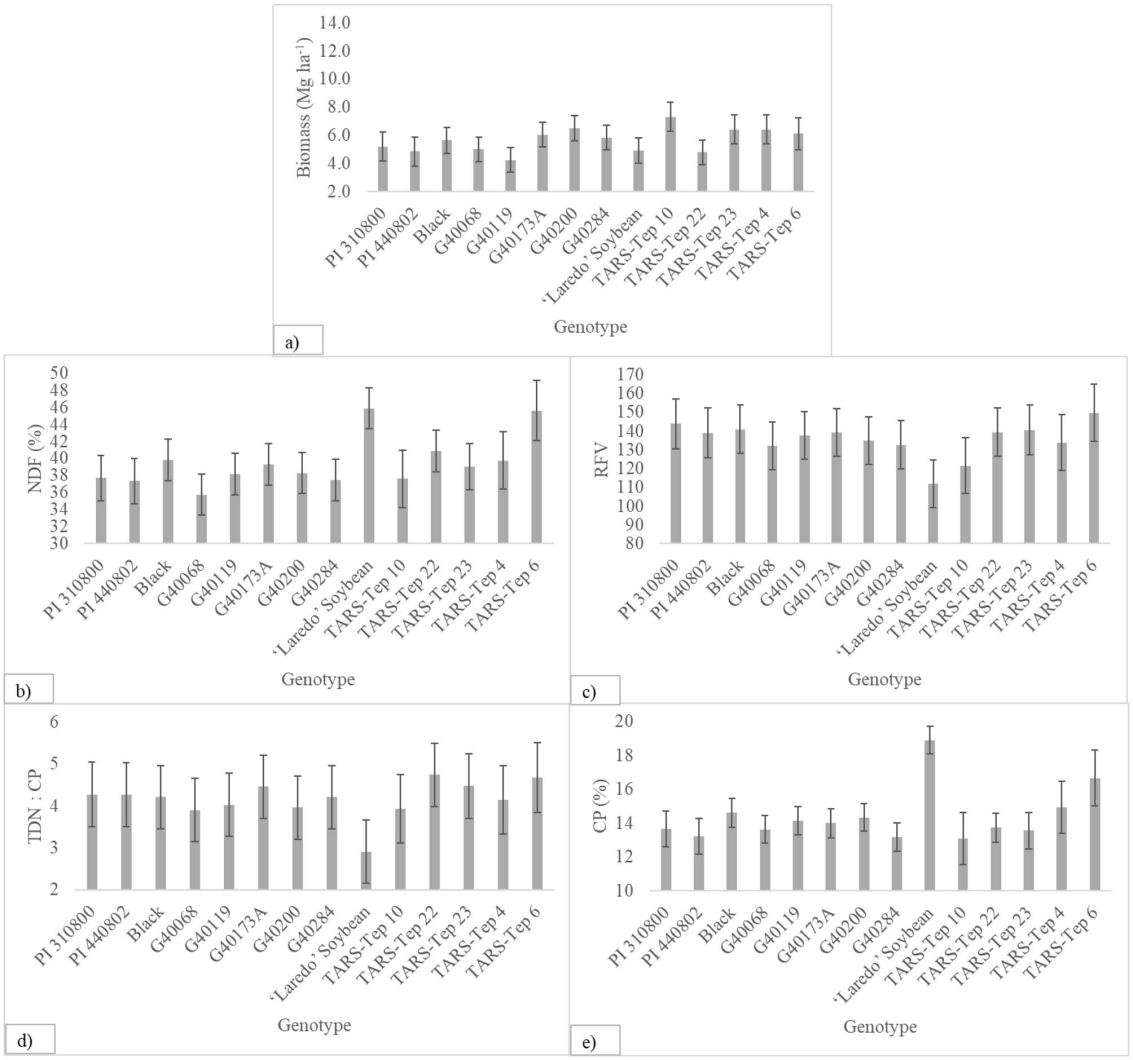
Yield and forage quality of 13 genotypes of tepary bean and one control (forage soybean) averaged across 7 cutting regimes at El Reno, OK during 2020 and 2021. Error bars show the standard error of the mean.

The genotype effect significantly influenced the response of biomass production, CP, NDF, RFV, and TDN:CP in tepary bean. Across all management regimes, average production of biomass ranged from 7.3 Mg ha^−1^ for TARS-Tep 10 to 4.3 Mg ha^−1^ for G40119 (Fig. 1a). However, no genotypes of tepary bean outperformed soybean (*p* > 0.05). Within genotypes of tepary bean, TARS Tep 10 produced significantly more biomass than G40119 (*p* ≤ 0.05). The NDF (cell wall) values for the genotypes G40068, G40119, G40200, G40284, PI 310,800, and PI 440,802 were lower than the control (*p* ≤ 0.05). This includes a 22.0% difference in NDF values between soybean (45.9%) and G40068 (35.8%) (Fig. 1b). All genotypes of tepary bean except G40068, G40284, TARS-Tep 4, and Tep 10 had significantly better RFV than soybean (*p* ≤ 0.05). RFV values ranged from 150 for TARS-Tep 6 to 112 for soybean (Fig. 1c). The TDN:CP ratios of all genotypes of tepary bean except G40200 and TARS-Tep 10 were significantly greater than soybean (*p* ≤ 0.05). The TDN:CP ratio was greatest for TARS-Tep 22 (4.6) and least for soybean (2.9) (Fig. 1d). The genotypes TARS Tep 4 (14.9%) and TARS Tep 6 (16.7%) had similar (*p* > 0.05) CP to soybeans (18.9%) (Fig. 1e). All other genotypes had lower CP values (14.6–13.1%).

### Cutting management

**Figure 2:**
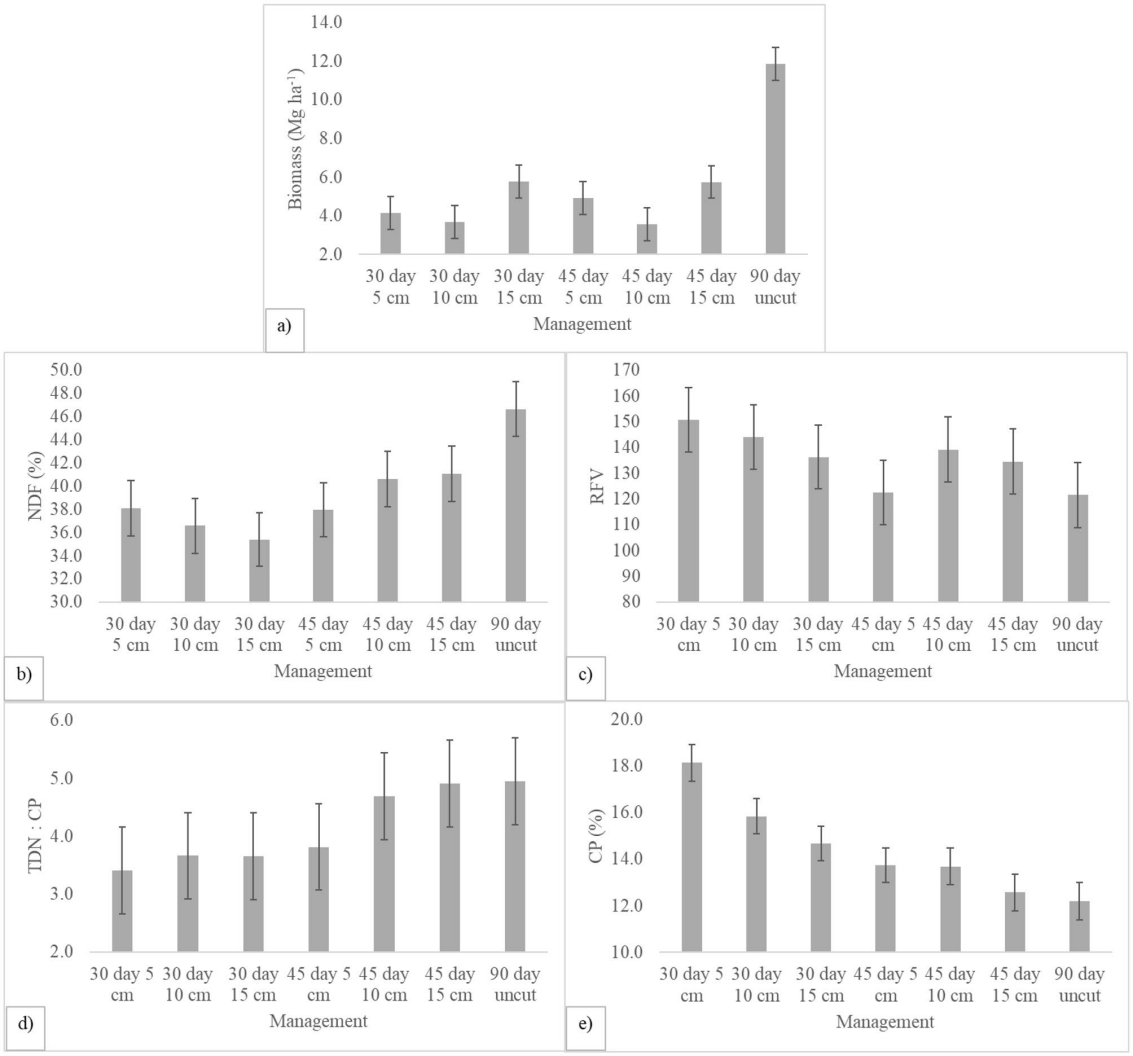
Average yield and forage quality of 13 genotypes of tepary bean and a control (forage soybean) under 7 cutting (across cultivars) regimes at El Reno, OK during 2020 and 2021. Error bars show standard error of the means.

Cutting management significantly affected all traits except IVTD and TDN. The 90-day cutting (control) produced significantly more biomass than the remaining management regimes (*p* ≤ 0.05), with an 8.3 Mg ha^−1^ difference between the uncut control and the 45-day × 10-cm cutting regime (Fig. 2a). All cutting regimes produced lower NDF than the uncut control (*p* ≤ 0.05), with values ranging from 46.7% for the uncut control to 35.4% for the 30-day × 15-cm regime (Fig. 2b). As with NDF, cutting improved ADF compared to the uncut control with a 22.9% difference in values between the uncut control and the 30-day × 15-cm regime (Fig. 2c). RFV was greater under the 5- and 10-cm × 30-daycutting regimes than the uncut control (*p* ≤ 0.05), with values ranging from 151 under the 30-day × 5-cm regime to 121 for the uncut control (Fig. 2d). The TDN:CP ratio ranged from 5.0 for the uncut control to 3.4 for the 30-day × 5-cm regime, a 31% difference (Fig. 2e). Cutting management produced significantly (*p* ≤ 0.05) greater CP than the uncut control for the 5- and 10-cm × 30-day cuttings. CP was greatest for the 30-day × 5-cm regime (18.1%) and least for the uncut control (12.2%). The product of combining biomass production and RFV was greatly impacted by cutting management. There was a 64% reduction between the 90-day cutting regime (1501) and the 45-day × 10-cm cutting regime (537).

## Discussion

This study evaluated forage biomass production and nutritive value of the underutilized grain legume, tepary bean, in response to different management regimes related to forage harvest. Producing forages with high nutritive value in the Southern Great Plains of the US is difficult under rainfed conditions due to unpredictable rainfall events or extended drought periods. Although the biomass production and nutritive value of forage produced by tepary bean and soybean were evaluated previously, their response to cutting management was unknown. The amounts of biomass produced by tepary bean in this study were similar to other studies conducted in Oklahoma and New Mexico^10,12^. Production by tepary bean was comparable to soybean, which indicates the two legumes have a similar ability to regrow after harvest. A study comparing responses to one cutting height at different points in time reported tepary bean produced more biomass than soybean at most time points^10^. In comparison, the uncut control in our study produced the greatest amounts of biomass. When comparing the other three management systems, the 15-cm cutting regimes generated the greatest biomass.

The lower levels of production by clipped tepary bean was related to effects of clipping on several physiological factors, such as; reduced nitrogen fixation, reductions in root growth and nutrient capture, lower amounts of labile carbohydrates available to support regrowth, and loss of photosynthetic tissues to capture carbon^19,20^. The 15-cm regimes may have outperformed the lower cutting heights because the latter lacked sufficient available carbon capture to support nodulation and nitrogen fixation^21^. The lower cutting heights likely also removed the organs required for regrowth by plants. In soybean, plants cut at 12 cm had greater regrowth than those cut at 7.5 cm due to a greater number of nodes and regrowth leaflets^22^. Annual legumes require enough remaining stem after cutting to provide meristematic tissues at locations where secondary stems and leaves attach to the primary stem for regrowth^20^. Lower cutting heights would remove stem that contains these tissues, reducing regrowth potential.

Environmental factors may also affect the impact of cutting height on legume regrowth. A Central Texas study compared nine annual legumes and reported total biomass production were 1.7 Mg ha^−1^ higher with a 15-cm cutting versus a 7.5-cm in one year, but the same treatments produced 0.9 Mg ha^−1^ less biomass in a different year^23^. This suggests growing conditions, particularly rainfall, may affect regrowth, and influence the height at which tepary bean should be cut. Genotype and cutting management both affected forage biomass and nutritive value in our study. Forage nutritive values were greater for some tepary bean genotypes than soybean, though individual traits varied. Generally, crude protein (CP) was higher in soybean, while tepary bean had greater digestibility due to lower NDF, though many genotypes of tepary bean performed similar to soybean.

Tepary bean in the SGP usually has a growing season of 90 days, though we observed differences in time of maturity (e.g., reproductive initiation, end-of-season leaf senescence) among genotypes of tepary bean, and between tepary bean and ‘Laredo’ soybean. These responses likely contributed to differences in NDF in this study. The low levels of NDF observed in tepary bean probably reflect a higher leaf to stem ratio. Baath et al. (2020a,b) reported leaf to stem ratios of tepary bean were equal to or greater than soybean, depending on the year. Further, tepary bean tends to senesce rather than abscise mature leaves as plant maturity increases^11,24^. In contrast, the leaves of soybean tend to abscise at the end of growing seasons. The lack of abscission in soybean with increased plant maturity was due to the longer growing season of soybean compared to tepary bean. The lower ADF and NDF values under the 30-day cutting regimes were likely due to a higher leaf to stem ratio in biomass than occurred under the other regimes.

Baath et al. (2020a) observed greater CP contents for tepary bean and soybean than was reported in the current study. However, frequent cuttings of legumes can reduce nodulation^21^ and ultimately they reduce the amount of CP in biomass. Nitrogen and CP contents in plant biomass usually decreases with age, but CP in soybean forage increases during pod filling^25–28^. Tepary bean has smaller seeds (10–18 g 100 seeds^−1^;^29^) than soybean (13.5–19.6 g 100 seeds^−1^;^30^), which may contribute to the lower CP in forage of tepary bean during later growth stages. The CP content of seeds of tepary bean ranges between 32.2 and 19.7%^31^ compared to 45.4 to 27.3% for soybean^32^.

The decline in CP in forage of tepary bean with plant age may be a drawback to using it as a hay crop compared to soybean. However, CP levels will depend on the timing of cuttings. Although the forage of tepary bean had less CP than soybean, the higher TDN:CP ratio was an improvement over soybean. However, the TDN:CP ratios of both tepary bean and soybean are inadequate to meet the needs of yearling stockers^33^. Therefore, the forage of tepary bean and soybean would both require a caloric energy supplement added to stocker diets to increase or sustain average daily gains. However, such supplementation is common for yearling cattle when grazing most forages. Overall, tepary bean is a promising forage or hay crop for the SGP, with better nutritive value (for some genotypes) than soybean during its early period of growth (June to September). In contrast, the longer growth cycle of forage soybean would give producers additional flexibility.

## Conclusion

Tepary bean is a viable alternative to forage soybean in the SGP, particularly under the hot, dry conditions that prevail during summers. During the 90-day period from June to September, some genotypes of tepary bean provided greater amounts of biomass and forage nutritive values than a forage soybean. The optimal management regime for tepary bean for forage was noted as one harvest applied at the end of growing seasons (90-day). Although some genotypes of tepary bean showed limited improvement in forage nutritive value with more-frequent cuttings, the benefit is outweighed by substantial losses in biomass production. Additionally, drought conditions may provide only one cutting to producers in the SGP due to limited regrowth. Breeding efforts to improve the forage characteristics and agronomic performance of tepary bean are required. Studies to determine the optimal planting rate/density are also needed to refine and expand the use of tepary bean as an alternative forage.

## Data Availability

Data is available USDA Ag Data Common (https://doi.org/10.15482/USDA.ADC/1528396).
